# Glycemic control and atrial fibrillation: an intricate relationship, yet under investigation

**DOI:** 10.1186/s12933-022-01473-0

**Published:** 2022-03-14

**Authors:** Andreas S. Papazoglou, Anastasios Kartas, Dimitrios V. Moysidis, Christos Tsagkaris, Stavros P. Papadakos, Alexandra Bekiaridou, Athanasios Samaras, Efstratios Karagiannidis, Marios Papadakis, George Giannakoulas

**Affiliations:** 1 First Department of Cardiology, AHEPA University Hospital, Aristotle University of Thessaloniki, St. Kiriakidi 1, 54636 Thessaloniki, Greece; 2 Athens Naval Hospital, Athens, Greece; 3 Faculty of Medicine, University of Crete, Heraklion, Greece; 4 First Department of Pathology, School of Medicine, National and Kapodistrian University of Athens, Athens, Greece; 5 University Hospital Witten-Herdecke, University of Witten-Herdecke, Heusnerstrasse 40, 42283 Wuppertal, Germany

**Keywords:** Diabetes mellitus, Atrial fibrillation, Comorbidity, Glycated hemoglobin, HbA1c

## Abstract

Atrial fibrillation (AF) and diabetes mellitus (DM) constitute two major closely inter-related chronic cardiovascular disorders whose concurrent prevalence rates are steadily increasing. Although, the pathogenic mechanisms behind the AF and DM comorbidity are still vague, it is now clear that DM precipitates AF occurrence. DM also affects the clinical course of established AF; it is associated with significant increase in the incidence of stroke, AF recurrence, and cardiovascular mortality. The impact of DM on AF management and prognosis has been adequately investigated. However, evidence on the relative impact of glycemic control using glycated hemoglobin levels is scarce. This review assesses up-to-date literature on the association between DM and AF. It also highlights the usefulness of glycated hemoglobin measurement for the prediction of AF and AF-related adverse events. Additionally, this review evaluates current anti-hyperglycemic treatment in the context of AF, and discusses AF-related decision-making in comorbid DM. Finally, it quotes significant remaining questions and sets some future strategies with the potential to effectively deal with this prevalent comorbidity.

## Introduction

Diabetes mellitus (DM) and atrial fibrillation (AF) constitute common chronic clinical entities. As of 2021, more than 530 million individuals worldwide live with DM; current trends suggest further rise in its global prevalence [1]. Similarly, AF, the most common cardiac arrhythmia, affects almost 40 million patients globally. The AF epidemic is expected to spread further in the next decades, along with the ever aging population [2]. Besides, DM and AF share common antecedents such as arterial hypertension, atherosclerosis and obesity [3, 4]. Hence, the AF-DM coexistence emerges as a global health burden with steadily mounting incidence. Major public health implications include the risk of major adverse cardiovascular (CV), cerebrovascular events, mortality, and escalating healthcare costs [5].

Although the epidemiology and implications of the AF and DM comorbidity seem to be well described in the current literature [6, 7], the precise underlying pathogenic mechanisms still remain an issue of debate [8]. Significant questions also remain regarding the effect of glycemic control on the development and clinical course of AF. These questions will be the focus of this mini review, following a summary on the epidemiologic and pathophysiological background of the comorbidity.

## The glycemic burden behind AF onset

### The epidemiologic interplay between AF and DM

Several population-based studies suggest that DM is an independent risk factor for AF development. In the early 1990s and after almost 4-decades of follow-up, the Framingham Heart study was first to suggest an independent association between DM and higher rates of AF incidence [9]. The accumulation of data from ensuing observational studies did not establish a direct causal relation between DM and AF, although it strengthened the notion that DM is an independent determinant for AF development [9–12]. The latter holds true for both type 1 and 2 DM, as well as pre-diabetes [13, 14]. Of note, the prevalence of DM varies to a significant extent among recent AF cohorts; from 9 to 32% [15–21]. The risk of AF seems to be correlated in a linear fashion with DM duration [17] and with glycemic control [higher glycated hemoglobin A1c (HbA1c) and DM duration over 20 years associated with elevated risk] [10, 12, 22].

Remarkably among diabetic individuals, female gender has been consistently associated with higher risk for AF when compared to male gender [10, 13, 23]. This was replicated by a machine-learning aided meta-analysis, whereby women with DM were 24% more likely to develop AF than men [24].

**Fig. 1:**
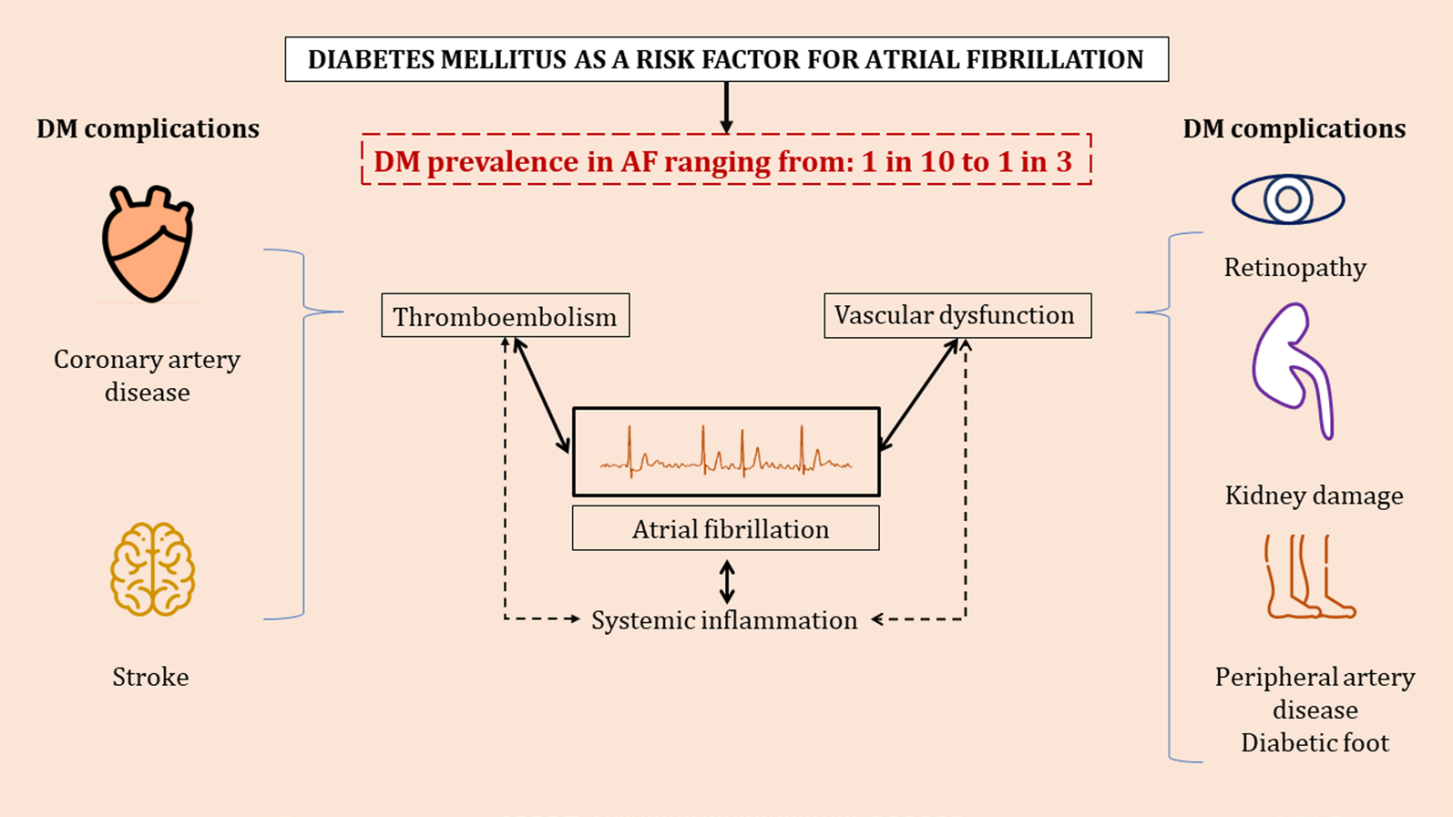
Association of diabetes mellitus with atrial fibrillation

Furthermore, the magnitude of association varies between DM and AF types; persistent or permanent AF is the most prevalent type in diabetic populations, according to a recent meta-analysis of 20 relevant studies [7]. The presence of DM may also enhance progression from paroxysmal to persistent AF [7, 25]. Moreover, the subjective symptoms of AF may be masked by DM-associated neuropathy and, therefore, delay diagnosis and treatment [26–28]. DM-induced autonomic neuropathy might depress the cardiac symptoms of an incident AF episode, not directly by impacting the conducting system, but rather by blunting the sensitivity of cardiac nerves [26, 29, 30]. Some investigators suggest that the abnormal central processing of afferent pain messages might also have a role to play in silent AF manifestation [25, 31]. In any case, AF-symptom masking in the setting of DM raises the question of whether these patients should be systematically screened for silent AF (Fig. 1).

### The underlying pathophysiological interlinks for DM-related AF

In this section we briefly outline the basic pathophysiological mechanisms linking DM with AF onset. This event is determined by metabolic alterations inherent to DM, including defects in haemostasis and fibrinolysis, increased angiogenesis, insulin resistance, and glucose intolerance, [32]. These changes result in activation of the renin–angiotensin–aldosterone system (RAAS). The latter exerts an atherogenic and pro-fibrotic stimuli on the cardiac muscle, forming the substrate for AF development [33].

#### Electrical-electromechanical remodeling

Inflammation is evident in DM and may underlie its pathophysiologic processes [34–36]. Oxidative stress, in the setting of chronic, subclinical inflammation, is another AF precipitant. Chronic inflammation increases the amount of reactive oxygen species (ROS) into the bloodstream. At the same time, the enzymes that degrade ROS fall in amount. This translates into fibrosis-related electromechanical changes in the atria [5]. Fibrosis and fat deposits in the atria are classic precursors and sustainers of AF. In DM, these pathologo-anatomical changes are the cause of the diminished voltages produced by the atria [37]. They also prolongate electrical conduction and disrupt the atrial excitation–contraction coupling [12, 38, 39]. Furthermore, they act as paracrine signaling molecules, namely cytokines, chemokines, adipokines that exacerbate AF. Diabetics undergoing electrophysiologic testing often exhibit proarrhythmic indices; shortened effective refractory period (ERP), ERP dispersion, slowed inter- and intra-atrial conduction velocity, spatially dispersed and heterogeneous in its slowing electrical conduction [40].

#### Structural remodeling

The two basic features of atrial structural remodeling are dilatation and fibrosis. In DM, diffuse interstitial fibrosis is initiated by the production of advanced glycation end products (AGEs) which upregulate the connective tissue growth factor [12]. The stiffening of the cardiac muscle promotes diastolic dysfunction of the left ventricle (LV) and left atrium (LA); the ensuing increase in left ventricular filling pressures and LA dilation promotes AF [5]. Experimentally, excessive myocardial accumulation of glycogen granules seems to be related to larger LA diameter, wider orifice and increased depth of the left atrial appendage, greater end-diastolic and end-systolic diameter, and lower E/A ratio [41]. Moreover, LV hypertrophy—a well-known risk factor and prognostic modifier of AF [42]—has been also associated with DM and abnormal glucose tolerance [36, 43].

#### Autonomic remodeling

DM has been linked with increased sympathetic and decreased parasympathetic activity of the cardiac muscle [42], which lead to decreased ERP. Vulnerability to AF is further promoted by the more heterogeneous distribution of the sympathetic innervation in the atria of diabetic individuals [12, 44].

## The potential role of AF in DM development

Apart from the fundamental aspects of DM and AF onset, the inverse relationship has been also hypothesized. AF is deemed to be associated with thromboembolism, microvascular dysfunction and systemic inflammation [45]. Consequently, organs and structures prone to the long-term systemic and vascular complications of AF, such as the pancreas, would likely develop dysfunction over time. This mechanism supports the less known assumption that AF in its own right may increase the risk of adult-onset DM [46].

## Relevance of glycated hemoglobin measurement in AF

### Glycated hemoglobin levels and AF onset

Preventing or delaying AF onset has drawn clinical interest during the last decade. Thereby, individuals could avoid or delay its fatal and non-fatal complications [47]. To that end, several prospective and retrospective studies have assessed associations between modifiable risk factors and the risk of AF. Two of those factors are considered to be the obesity and the individual glycemic control, as expressed through body mass index, fasting serum glucose or HbA1c values. Considering the reciprocal relationship between obesity and glycemic control, one should be cautious to draw definite conclusions on their distinct association with AF development. Specifically, obesity per se has been significantly linked with new-onset AF [48–50], while a recent meta-analysis revealed a 10% increased risk of AF per 20 mg/dl increase in blood glucose [14]. Another meta-analysis considering both diabetic and healthy individuals yielded that a 1% increase of HbA1c was linked with a 13% increased risk of AF occurrence [51]. HbA1c levels higher than 6.3% were significantly associated with an increased risk of AF incidence among 352,325 individuals from 14 studies, irrespective of DM history [52]. Another recent study including over 2 million participants with and without DM, showed that there was a significantly excess risk of AF, linearly correlated with increasing HbA1c levels and albuminuria stages [10]. Micro- or macro-albuminuria have been already proposed as markers of deranged HbA1c levels, and tight glycemic control has been shown to reduce the incidence of albuminuria in DM [53, 54]. Additionally, Hsu et al. identified that the long-term visit-to-visit glycemic variability was independently associated with the development of new-onset AF in 27,246 subjects with type 2 DM [55]. Consequently, serum HbA1c levels, especially in the range of prediabetes or diabetes, may be viewed as a potential predictive biomarker of AF incidence.

### Glycated hemoglobin levels and AF clinical course

Apart from predicting AF, the use of HbA1c has also been proposed as a monitoring tool in AF and AF-related complications. Poor glycemic control and glycemic fluctuations have previously proved to be strong predictors for both micro- and macro-vascular disease, as markers of ambient hyperglycemia [56]. Glycemic variability seems to impose greater vascular damage, autonomic dysfunction and cardiomyopathy than chronic stable hyperglycemia [57].

#### Risk of stroke

Cerebral microvascular dysfunction may be apparent in adults with DM and AF as a DM-related complication [58]. However, strokes are the tip of the iceberg of cerebrovascular adverse events in AF. As indicated by the presence of DM as a dichotomous variable in CHA_2_DS and CHA_2_DS_2_-VASc scores [59], DM has been correlated with higher risk for stroke in most studies of AF patients [60–63] even though other included categorical parameters such as female gender and vascular disease proved to have stronger associations [64]. The tendency for stroke in DM probably occurs as a result of enhanced thrombin generation and impaired fibrinolysis in DM [65, 66]. Patients with long-term or insulin-dependent DM are reported to carry an especially increased rate of ischemic stroke [27, 67, 68], and might benefit more from oral anticoagulation, even in the absence of other major risk factors for stroke [5].

However, the glycemic status of these patients seems to be the cardinal parameter determining stroke risk, with HbA1c levels above 6.5% signaling a higher risk early in the course of DM [69]. In 2015, Saliba et al. demonstrated that the addition of HbA1c to the CHA_2_DS_2_-VASc score—beyond the dichotomous DM presence—improved the predictive accuracy of the model [70]. Chan et al. found an association of HbA1c levels above 6.5% with increased risk of thromboembolism to also exist in non-diabetic patients with AF [71]. The latter study also suggested that non vitamin K oral anticoagulants (NOACs) were more effective than warfarin for preventing thromboembolism across broad HbA1c categories [71]. Therefore, earlier and tighter control of HbA1c, as well as use of NOACs over warfarin could help mitigate the increased risk of thromboembolism in AF patients with or without DM.

Of note, some recent studies did not yield increased risk of stroke in AF patients with poor or intermediate glycemic control [20, 21, 67, 72, 73]. A potential reason proposed to account for that discordance might be the underlying mechanism for stroke in patients with AF, which is mainly atrio-embolic and not so often due to underlying atherosclerosis [67, 74, 75]. These studies suggested that duration of DM might be a more significant prognostic parameter than glycemic control among AF populations, due to enhanced thrombin generation, prothrombotic fibrin clot properties, and impaired fibrinolysis in the first case [65, 66].

#### Risk of bleeding

Bleeding events, be it major or minor, occur more frequently in AF, given the anticoagulation therapeutic schemes needed [76]. Some studies suggest that DM might further increase the risk of bleeding during anticoagulation therapy [22, 77]. Specifically, Karayiannides et al. found that bleeding complications were increased in DM after adjustment for other comorbidities and medications. No substantial difference was observed among various anti-hyperglycemic medication groups. The authors concluded that the rate of bleeding complications could be likely improved with the increased use of NOACs in the context of AF [77]. On the contrary, other observational analyses of AF populations did not reveal significantly elevated risk of bleeding in patients with comorbid DM [16, 20, 21, 68]. Subgroup analyses taking into consideration the duration of DM or patients’ glycemic control also failed to yield significant differences. Hence, the totality of the evidence concurs with the absence of DM or HbA1c levels among the contributing parameters of the HAS-BLED score.

#### Risk of mortality and cardiovascular hospitalizations

DM is considered to be a burden in the clinical course of AF. There is a well-established link between DM or poorly regulated blood glucose levels and increased mortality in patients with AF [6, 21, 24, 63, 77]. In particular, a positive linear correlation exists between HbA1c and mortality rates. Levels above 7.6% and below 6.2% have been proposed as markers of increased and decreased mortality, respectively [21]. Other observational studies on diabetic populations without AF have demonstrated that both extremely low and high HbA1c levels may herald increased mortality rates (J or U shaped curves) [78, 79]. Yet, these curves have not been encountered in AF populations. Regarding survival rates of AF patients without DM, a non-significant association of increased mortality with increased HbA1c levels has been observed [24].

Furthermore, AF patients with DM usually have higher incidence of hospitalizations, as compared with patients without DM [21, 80]. Poor or intermediate glycemic control (HbA1c > 7%) in DM correlates with more frequent AF-related hospitalizations. Notwithstanding the increased rates of hospitalization in patients with DM, rates of in-hospital mortality in AF seem not to be negatively affected by the presence of comorbid DM [19, 80]. Nevertheless, more observational studies are warranted to conclude on the impact of HbA1c on the survival of diabetic and non-diabetic AF populations.

### Glycated hemoglobin levels and AF ablation

**Fig. 2:**
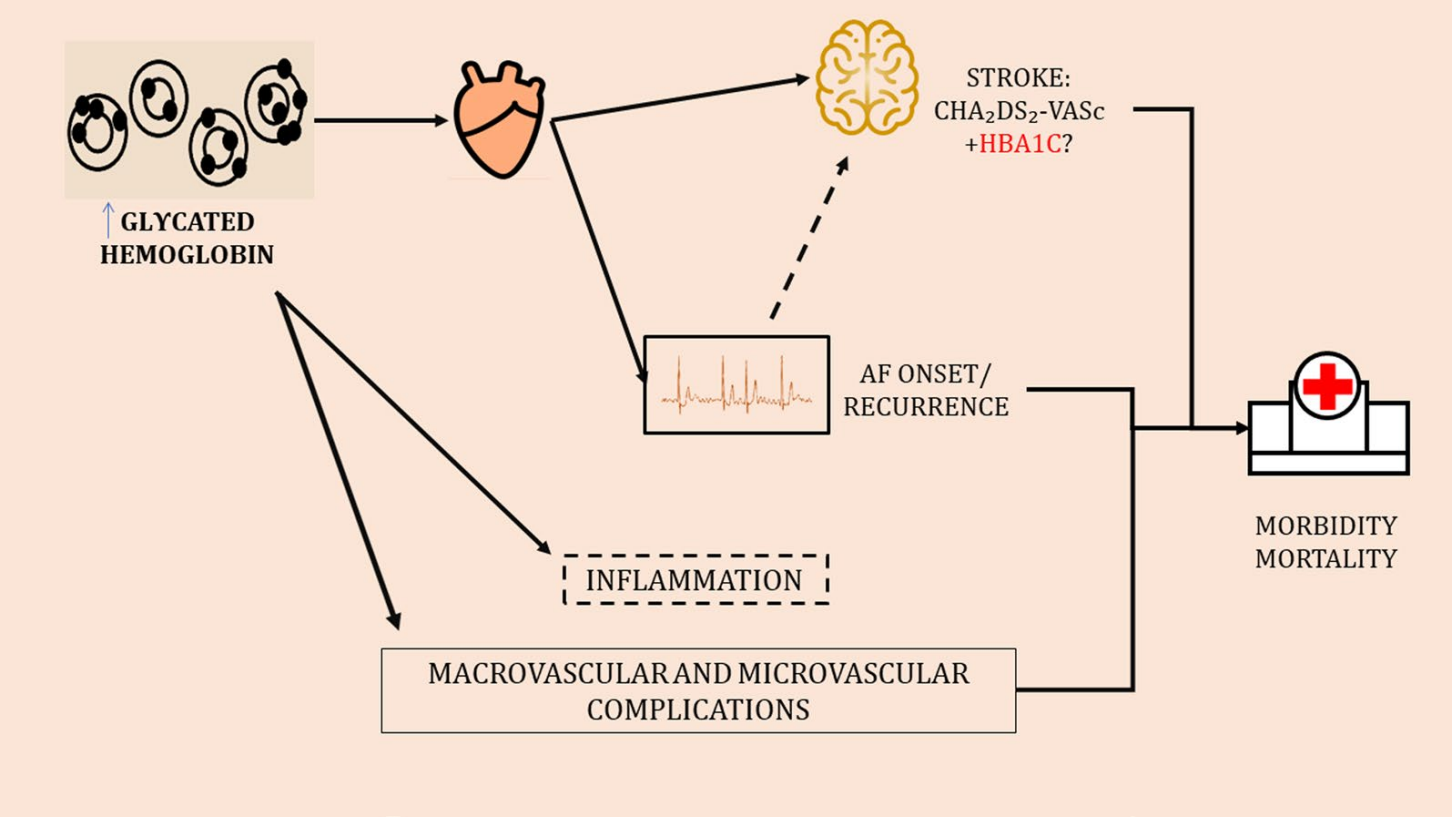
Glycemic control as a risk factor for atrial fibrillation-related adverse outcomes

Catheter ablation has emerged as an interventional strategy conjunctive with or alternative to antiarrhythmic drugs with the potential to treat abnormal heart rhythm with comparable or even superior outcomes. Based on recent observational studies, there is no significant difference in the rates of peri-procedural complications among DM and non DM patients [81]. Additionally, efficacy and safety of cryo-ablation has been shown comparable to radio-frequency ablation in both groups [15, 33]. Nevertheless, arrhythmia-free survival has been reported to be significantly lower among patients with DM [81, 82]. Younger age and aggressive modification of lifestyle risk factors, such as weight loss and optimization of glycemic control are important factors related to peri-procedural outcomes and arrhythmic recurrences [83, 84]. A study of 298 AF patients with DM undergoing catheter ablation found that improvement of HbA1c levels by more than 10% in the 12 months prior to ablation was independently associated with 30% decreased risk of AF recurrence [85]. Lu et al. also suggested that HbA1c of less than 6.9% could account for greater success rates of ablation [86]. Last, the use of metformin has been associated with a significant decrease in post-ablation AF recurrence after adjustment for pre-procedural glycemic control [87] (Table 1, Fig. 2).

**Table 1:** Association of glycated hemoglobin levels with atrial fibrillation-related outcomes

Condition assessed	Study	Impact of glycemic control	Study type
AF development	Αune, D. et al., 2015 [14]	Increased incidence of AF in diabetic patients:	Meta-analysis
		RR: 1.30, 95% (CIs 1.03–1.66)	
		Increased incidence of AF in diabetic patients per 20 mg/dl increase of blood glucose:	
		RR: 1.11 (95% CIs 1.04–1.18)	
	Zhao H. et al., 2020 [52]	Increased incidence of AF in diabetic and non-diabetic patients per 1% increase of HbA1c:	Meta-analysis
		RR: 1.16 (95% CI 1.07–1.27)	
	Qi W. et al., 2017 [51]	Increased incidence of AF in diabetic patients per 1% increase of HbA1c:	Meta-analysis
		RR: 1.13, 95% (CIs 1.09–1.18)	
	Huxley, R.R. et al., 2012 [88]	Increased incidence of AF in diabetics with poor glycemic control:	Original research
		HR: 1.13, (95% CIs 1.07–1.20) per 1% point increase of HbA1c	
	Iguchi, Y. et al., 2012 [89]	Elevating HbA1c associated with higher prevalence of AF:	Original research
		OR: 1.18 (95% Cis 1.09–1.28)	
	Dublin, S. et al., 2010 [90]	Higher risk for developing AF in individuals with worse glycemic control compared to those without DM:	Original research
		**HbA1c ≤ 7**: adjusted OR: 1.06 (95% CI 0.74–1.51)	
		**7 < HbA1c < 8**: adjusted OR: 1.48 (95% CI 1.09–2.01)	
		**8 < HbA1c < 9**: adjusted OR: 1.46 (95% CI 1.02–2.08)	
		**HbA1c > 9**: adjusted OR: 1.96 (95% CI 1.22–3.14)	
	Fatemi, O. et al., 2014 [91]	Intensive glycemic control not affecting the risk of AF incidence:	Original research
		Incident AF occurred in 159 patients (1.58%) over the follow-up period at a rate of 5.9/1,000 person-years in the intensive-therapy group (**targeting at HbA1c < 6.0%**), and at a rate of 6.37/1,000 person-years in the standard-therapy group (**targeting at** 7% < **HbA1c < 7.9%**) (p = 0.52)	
	Ahmadi, SS. et al., 2020 [10]	Increased incidence of AF in diabetic individuals compared with age- and sex-matched controls	Original research
		aHR: 1.28 (95% CIs 1.26–1.30)	
Risk of stroke	Saliba, W. et al., 2015 [70]	Increased risk of stroke among AF patients with higher HbA1c levels in comparison with patients without DM:	Original research
		**HbA1c < 6.35%** HR: 1.04, 95% CI 0.83–1.30	
		**HbA1c 6.35–6.90%** HR:1.14, 95% CI 0.92–1.42)	
		**HbA1c 6.91–7.70%** HR: 1.46, 95% CI 1.19–1.79	
		**HbA1c > 7.70%** HR: 1.63, 95% CI 1.33–2.00	
		Among AF patients with DM, HR: 1.17 (95% CI 1.09–1.26) for every 1% increment in HbA1c	
		The AUC was 0.585 for the CHA2DS2-VASc score, which increased to 0.604 when HbA1c was included in the model (p = 0.038)	
	Fangel MV. et al., 2019 [69]	Increased risk of stroke among AF patients with higher HbA1c levels	Original research
		• aHR: 1.49 (95% CIs: 1.09–2.05) for patients with **HbA1c = 49–58 mmol/mol** compared to HbA1c ≤ 48 mmol/mol	
		• aHR: 1.59 (95% CI 1.13–2.22): for patients with **HbA1c > 58 mmol/mol** compared to HbA1c ≤ 48 mmol/mol	
	Chan, YH. et al., 2020 [71]	Increased risk of ischemic stroke/thromboembolism among AF patients with higher HbA1c levels:	Original research
		Compared with patients with an HbA1c level of < 5.4%, the risk significantly increased when HbA1c levels were higher than 6.5%	
		• aHR: 1.20 (95% CIs 1.00–1.43) for **HbA1c level of 6.5–6.9%**	
		• aHR: 1.32 (95% CIs 1.11–1.57) **for HbA1c level of 7.0–7.9%**, and	
		• aHR: 1.48 (95% CI 1.25–1.76) for **HbA1c level of ≥ 8.0%**	
Risk of mortality or hospitalizations	Papazoglou AS. et al., 2021 [21]	Risk of all-cause mortality among diabetic AF patients depending on HbA1c levels:	Original research
		HbA1c levels **above 7.6% and below 6.2%** have been proposed as markers of increased and decreased mortality, respectively	
	Kanellopoulou K. et al. 2018 [24]	Increased risk of all-cause mortality among diabetic AF patients with higher HbA1c levels:	Original research
		. The mortality for AF patients with stroke history is increased with the increase of HbA1c in patients with DM in a statistically significant manner (p < 0.001). A non-significant increase in mortality was observed in patients without DM. (p = 0.22)	
	Selvin E. et al. 2010 [78]	J-shaped association between HbA1c and the risk of all-cause mortality among individuals without DM:	Original research
		• **HbA1c < 5.0%:** aHR: 1.48 (95% CIs1.21–1.81)	
		• **HbA1c = 5.0 to < 5.5% (reference):** aHR: 1.00 (95% CIs 1.00–1.00)	
		• **HbA1c = 5.5 to < 6.0%:** aHR: 1.19 (95% CIs 1.05–1.35)	
		• **HbA1c = 6.0 to < 6.5%:** 1.61 (1.35–1.91)	
		• **HbA1c ≥ 6.5%:** aHR: 1.71 (95% CIs 1.30–2.25)	
	Li W. et al., 2016 [79]	J-shaped association between HbA1c and the risk of all-cause mortality among patients with DM:	Original research
		• **HbA1c < 6.0%:** aHR: 1.06 (95% CIs0.92–1.24)	
		• **HbA1c = 6.0 to < 6.9% (reference):** aHR: 1.00	
		• **HbA1c = 7.0 to < 7.9%:** aHR: 1.10 (95% CI 0.92–1.30)	
		• **HbA1c = 8.0 to < 8.9%:** 0.93 (95% CI 0.75–1.16)	
		• **HbA1c = 9.0 to < 0.9%:** 1.26 (95% CI 1.01–1.58)	
		• **HbA1c = 10.0 to < 10.9%:** 1.18 (95% CI 0.93–1.51)	
		• **HbA1c ≥ 11.0%:** aHR: 1.31 (95% CI1.08–1.60)	
AF ablation success	Lu, Z.H. et al., 2015 [86]	Higher levels of HbA1c associated with increased risk of AF recurrence of atrial tachyarrhythmia in DM patients undergoing catheter ablation:	Original research
		• **HbA1c < 6.9%:** success rate of ablation was 69.0%	
		**HbA1c ≥ 6.9%:** success rate of ablation 46.8% (p = 0.004)	
		• HbA1c was independent predictor of recurrent atrial tachyarrhythmia: aHR: 1.22, 95% CI 1.02–1.47	
		• **HbA1c cut-off value of ≥ 6.9%** predicted AF recurrence with 55.0% sensitivity and 67.4% specificity (AUC = 0.634)	
	Donnellan E. et al., 2019 [85]	Better outcomes of AF catheter ablation with improvement of pre-procedural HbA1c levels:	Original research
		Improvement of HbA1c levels 12 months prior to ablation by more than 10% was independently associated with 30% decreased risk of AF recurrence. 68.75% of patients with **HbA1c > 9%** at the time of ablation developed recurrent AF, compared with 32.4% of those with **HbA1c < 7%** (p < 0.0001)	
	Stout KM. et al., 2021 [92]	Increased risk of recurrent atrial arrhythmias and cardiovascular hospitalizations following AF ablation with higher HbA1c levels:	Original research
		HR: 1.57 (95% CIs 1.02–2.36)	

Bold values represent specific HbA1c cut-off values, as assessed by each included study

HbA1c, glycated hemoglobin A1c; DM, diabetes mellitus; AF, atrial fibrillation; AUC, area under the receiver operating characteristic curve; RR, risk ratio; OR, odds ratio; (a) HR, (adjusted) hazard ratio, CI, confidence interval

## Diabetic treatment in AF

Clarifying the impact of anti-hyperglycemic drugs on coexisting AF is still hindered by vague evidence. The randomized, double-blind investigation of 10,082 patients with DM from the ACCORD cohort yielded that intensive glycemic control (targeting at HbA1c < 6.0%) did not affect the rate of new-onset AF compared to HbA1c 7–7.9% [91]. However, most recently conducted studies showed that the detrimental effects of glycemic fluctuations (hypoglycemic episodes) may have offset theoretical advantages of intensive glycemic control regarding AF incidence and progression [93, 94]. Specifically, insulin administration seems to be associated with an increased risk of AF-related complications [93, 95, 96]. Either due to low blood glucose levels per se or through activation of sympatho-adrenal response the refractory period is shortened and, hence, hypoglycemia acts as a trigger of paroxysmal and more progressive forms of AF [97]. Therefore, such fluctuations should be avoided in subjects with comorbid AF who require more individualized approaches to diabetic care with the aim of keeping blood glycemic levels low but stable.

Besides insulin, dipeptidyl peptidase 4 inhibitors (gliptins) have been also correlated with increased risk of AF onset [95]. Available aggregate data suggest that glucagon-like peptide-1 (GLP-1) receptor agonists (exenatide, liraglutide, lixisenatide, dulaglutide, or semaglutide) are not associated with AF onset, with the only possible exception of albiglutide [98, 99]. Even if these data do not establish causation, recent studies showed that biguanides (metformin) [100], thiazolidinediones (pioglitazone) [100], secretagogues (sulfonylurea) [95] and sodium-glucose cotransporter 2 (SGLT-2) inhibitors (empagliflozin, dapagliflozin) [101, 102] have a decreased risk of AF recurrence or new onset AF. More specific, the use of SLGT-2 inhibitors has been associated with decreased rates of heart failure related hospitalization or CV death in AF and, therefore, could be utilized as a cardioprotective medication in AF patients with DM being at high CV risk [27]. Finally, the preventive role of thiazolidinediones for atrial remodeling and the benefits of pioglitazone as upstream therapy after catheter ablation are further noteworthy findings of the existing literature [12, 103]. However, thiazolidinediones have been linked with fluid retention, and should therefore be avoided in diabetic patients with heart failure [104].

## Anticoagulation and AF-related decision-making in comorbid DM

In general, the management of AF in patients with comorbid DM should comply with the simple Atrial fibrillation Better Care (ABC) holistic pathway (‘A’ Anticoagulation/Avoid stroke; ‘B’ Better symptom management; ‘C’ Cardiovascular and Comorbidity optimization), as proposed by the 2020 ESC Guidelines for the diagnosis and management of AF [105, 106]. Concerning anticoagulation (“A”), the 2019 ESC Guidelines on diabetes, pre-diabetes, and CV diseases recommend that it should be initiated in all AF patients with DM [107]. Clinicians should also take into account patients’ glycemic control to predict more accurately their risk of future thromboembolic events [70]. However, chronic kidney disease, a common DM complication, constitutes a further treatment challenge. It affects the choice of the anticoagulation strategy since NOACs are partially eliminated through the kidney. Compared to vitamin K antagonists, NOAC therapy seems beneficial to a similar extent in DM and no DM alike; there is no effect modification of DM on the relative reduction with NOACs in the risk of stroke, CV mortality, bleeding, and progression of renal function impairment [107, 108].

With regard to better (“B”) AF-related symptom management, the presence of DM and/or increased HbA1c levels do not seem to affect the efficacy of pharmaceutical rate or rhythm control treatment strategies; yet electrical cardioversion and AF-ablation procedures seem to have higher rates of short- and long-term failure [109]. In the ABC pathway, the optimal management of comorbidities (“C”) is of utmost importance to ameliorate the outcomes of AF, and, therefore, DM requires optimal guideline-directed management [106]. The clinician should be aware, of course, of the severe CV morbidity burden that DM carries on, and be prepared to prevent heart failure by focusing on its early signs and initiating with SGLT-2 inhibitors particularly in patients at higher CV risk [110, 111].

## Remaining questions and future strategies

Τhere is still paucity of outcomes research on a national scale for this comorbidity, which could help in updating the available guidelines on the decision-making when both conditions coexist [105]. Experts across specialties including cardiology and endocrinology need to work together in future and encapsulate the optimal approach for prevention and management of this comorbidity whilst acknowledging DM as a CV disease. More specifically, it might be important to establish optimal, “arrhythmia safe’’ HbA1c cut-off levels, once further related research is performed and aggregate data are collected, reviewed and/or meta-analyzed. The integration of HbA1c levels as a dichotomous variable in the CHA_2_DS_2_-VASc anticoagulation decision-making tool might be a robust and groundbreaking strategy to consider for validation in future observational studies. A multidisciplinary approach targeting at the development of screening programs for silent AF might also warrant the consideration of HbA1c levels as well; however relevant data are scarce.

To that end, recently proposed metabolic biomarkers beyond HbA1c, such as AGEs, advanced oxidation protein products, thiobarbituric-acid reacting substances, fructosamine, and 1,5-anhydro-d-glucitol, might evolve into surrogate biomarkers for the risk stratification of AF populations with comorbid DM. Specifically, serum levels of AGEs have been inversely associated with optimal outcomes in AF patients undergoing pulmonary vein isolation [112], while lower 1,5-anhydro-d-glucitol levels have been linked with worse prognosis in patients with CV disease [113–117]. Higher fructosamine levels, reflecting acute oxidative stress, have been suggested as predictive biomarkers of both micro- and macro-vascular outcomes with similar magnitude of association to that of HbA1c [118–120]. Hence, these associations might occur in AF patients with DM as well, which warrants further investigation.

## Conclusions

The inter-relationship between DM and AF is complex and reciprocal. DM increases the risk of developing AF and has been associated with increased CV morbidity burden and mortality rates. Glycemic status, expressed via HbA1c levels, seems to play a substantial role in various instances of AF care; risk of AF, stroke prevention, catheter ablation success and overall survival. To that end, an integrated approach and a more individualized management of AF and DM are required to reduce the risk of AF- or DM-related complications and achieve optimal patient outcomes.

## Data Availability

Not applicable.

## Abbreviations

AF: Atrial fibrillation
DM: Diabetes mellitus
HbA1c: Hemoglobin A1c
CV: Cardiovascular
